# Epinephrine as a potential driver of oral lichen planus pathogenesis

**DOI:** 10.1080/19768354.2025.2588914

**Published:** 2025-11-17

**Authors:** Yu Gyung Kim, Kun-Hwa Kang, Hyo-Jin Song, Won Jung, Sungil Jang, Jin-Seok Byun, Do-Yeon Kim

**Affiliations:** a Department of Pharmacology, School of Dentistry, Kyungpook National University, Daegu, 41940, Republic of Korea; b Department of Oral Medicine, School of Dentistry, Kyungpook National University, Daegu, 41940, Republic of Korea; c Department of Oral Medicine, Institute of Oral Bioscience, School of Dentistry, Jeonbuk National University, Jeonju, 54907, Republic of Korea; d Department of Oral Biochemistry, Institute of Oral Bioscience, School of Dentistry, Jeonbuk National University, Jeonju, 54907, Republic of Korea; e Department of Pharmacology, School of Dentistry, Brain Science and Engineering Institute, Kyungpook National University, Daegu, 41940, Republic of Korea

**Keywords:** Oral lichen planus, epinephrine, oxidative stress, STAT3, cell death

## Abstract

Oral lichen planus (OLP) is a chronic inflammatory condition characterized by CD8+ T cell-mediated apoptosis of oral epithelial cells. While psychological stress has been implicated in OLP pathogenesis, the underlying mechanisms remain unclear. This study explores the role of epinephrine, a primary stress-related catecholamine, in OLP progression. We found that high concentrations of epinephrine induce cytotoxicity in oral keratinocytes, marked by reduced cell viability and increased DNA damage. High-dose epinephrine also elevates oxidative stress by downregulating antioxidant proteins SOD2 and SESN2. Additionally, it activates the STAT3 signaling pathway through both alpha- and beta-adrenergic receptors. Furthermore, epinephrine increases levels of HMGB1 and extracellular ATP, key damage-associated molecular patterns (DAMPs) that could perpetuate chronic inflammation in OLP. These findings suggest that stress-induced epinephrine may exacerbate OLP by promoting oxidative stress, epithelial damage, and immune activation. Given the increased vascularization in OLP lesions, epinephrine’s effects may be amplified in affected tissues. Understanding the link between stress and OLP pathogenesis could provide new therapeutic targets for managing this condition.

## 1. Introduction

Oral lichen planus (OLP) is a chronic or relapsing inflammatory condition characterized by CD8+ T cell-mediated apoptosis of oral epithelial cells. Lesions commonly present bilaterally on the buccal mucosa, tongue, and lips, with less frequent involvement of the palate and floor of the mouth (Krupaa et al. 2015). A recent meta-analysis research reported an overall estimated pooled prevalence of OLP to be 0.89% among the general population (Li et al. 2020), with a higher prevalence observed in non-Asian countries, among women, and in individuals over 40 years of age (Li et al. 2020). Patients with OLP often experience heightened sensitivity to spicy, acidic, or hot foods, along with symptoms such as pain, bleeding, and gingivitis. Severe cases may lead to secondary infections, scarring, and nutritional deficiencies due to eating-related discomfort, as well as an increased risk of oral cancer. Additionally, OLP patients exhibit significantly higher levels of stress, anxiety, and depression, leading to a diminished quality of life compared to non-OLP individuals (Chaudhary 2004).

Histopathologically, OLP is distinct from other oral mucosal lesions. The epithelium may exhibit hyperkeratosis (excessive keratinization) or parakeratosis (incomplete keratinization), depending on lesion type (Abe et al. 2020). A hallmark feature is the presence of a dense, band-like infiltrate of CD8+ T cells beneath the epithelium, contributing to chronic inflammation (Muller 2017). Eosinophilic, apoptotic keratinocytes located at the epithelial-connective tissue interface form Civatte bodies, which serve as a diagnostic marker for OLP. Persistent damage to the basal epithelial layer ultimately results in basal cell degeneration.

OLP is a multifactorial disease with an autoimmune basis, influenced by genetic predisposition and environmental triggers. Due to a wide range of external triggers, including mechanical trauma (Köbner phenomenon), dental materials (e.g. amalgam, gold), medications (e.g. NSAIDs, beta-blockers), or bacterial/viral infections, understanding the precise etiology of OLP is still challenging (Gupta and Jawanda 2015). While previous studies suggest that the inflammatory microenvironment plays a role in OLP development, inflammation alone does not appear sufficient to trigger disease onset (Kim et al. 2025). Given that inflammatory stimuli persist in the oral cavity, the episodic nature of OLP flares suggests additional contributing factors.

Mounting evidence supports a strong link between psychological stress and OLP. More than half of OLP patients report experiencing exceptionally high levels of stress, anxiety, or depression at the time of disease onset (Burkhart et al. 1996; Kim et al. 2023). Under the stress condition, cortisol and catecholamines (i.e. epinephrine and norepinephrine) play crucial roles. However, although still controversial, studies on cortisol revealed that patients with OLP did not differ from the controls in the level of cortisol (Rodstrom et al. 2001; Girardi et al. 2011). Instead, recent study showed that 3,4-dihydroxymandelic acid, a major metabolite of the catecholamines, is clearly upregulated in OLP patients, mirroring an increase in the release of epinephrine and norepinephrine (Yang et al. 2020). Notably, epinephrine, but not glucocorticoids, has been shown to induce apoptosis in epithelial cells (Sibayan et al. 2000). This raises the intriguing possibility that catecholamines may influence keratinocyte homeostasis and contribute to OLP pathogenesis, warranting further investigation.

## 2. Materials and methods

### 2.1. Cell culture

The HOK-16B human oral keratinocytes (RRID: CVCL_B405) were maintained in keratinocyte growth medium (PromoCell, Germany, cat no. C-20011) including supplementary growth factors and 150 μM CaCl_2_ in a humidified atmosphere containing 5% CO_2_ at 37°C.

### 2.2. Clinical samples

Total seven randomized Korean patients with OLP (mean ± s.d. age, 57.25 ± 11.08 years) who visited the Department of Oral Medicine, Jeonbuk National University Hospital were enrolled in the study with informed consent. Buccal mucosa was taken by incisional biopsy from each OLP patient, and the tissue was then divided into two parts: (1) the ‘non-ulcerative (normal) control,’ which exhibited no signs of white reticulation, red erosion, or ulceration, and (2) the ‘ulcerative lesion,’ which displayed clear ulceration and severe erosion. All the patients fulfilled the clinicopathological criteria of ulcerative OLP (van der Meij and van der Waal 2003). The following diseases and systemic conditions were excluded, such as uncontrolled hypertension, diabetes, pregnancy, autoimmune diseases including psoriasis and rheumatoid arthritis, other oral mucosal infectious diseases including oral candidiasis and herpes virus infection, and oral cancers. This study was approved by the Institutional Review Board of Jeonbuk National University Hospital (CUH 2021-02-044-008).

### 2.3. Protein preparation and immunoblot analysis

Cells were disrupted directly with Laemmli buffer (60 mM Tris-HCl (pH 6.8), 2% (*w/v*) sodium dodecyl sulfate (SDS), 10% (*v/v*) glycerol and 0.02% (*w/v*) bromophenol blue) before being sonicated and heat-denaturated for immunoblotting. Lysates were fractionated by SDS-polyacrylamide gel electrophoresis (SDS-PAGE) and proteins were transferred onto a polyvinylidene fluoride membrane. After blocking with 5% non-fat dried milk in Tris-buffered saline with Tween™ 20 (TBST) (10 mM Tris, pH 8.0, 150 mM NaCl and 0.5% Tween 20) for 30 min, membranes were incubated with antibodies against phospho STAT3 (1:1000, Cell signaling, Danvers, MA, USA, cat no. 9145), total STAT3 (1:1000, Cell signaling, cat no. 8768), β ACTIN (1:5000, Sigma Aldrich, cat no. A5316), phospho ERK (1:1000, Thermo Fisher Scientific, Waltham, MA, USA, cat no. MA5-15174), total ERK (1:1000, Cell signaling, cat no. 4695), phospho AKT (1:1000, Cell signaling, cat no. 4058), total AKT (1:1000, Cell signaling, cat no. 9272), BCL2 (1:1000, Bioworld Technology, China, cat no. BS1511), SOD2 (1:1000, Cusabio, Houston, TX, USA, cat no. CSB-PA022398LA01HU), and SESN2 (1:1000, Abcam, Cambridge, UK, cat no. ab178518) overnight at 4°C. Immunoreactive signals were detected with D-Plus™ ECL Femto system (Dongin LS, South Korea) or Clarity™ Western ECL Substrate (Bio-rad, Hercules, CA, USA). Densitometry was performed using ImageJ software.

### 2.4. Immunofluorescence

For immunofluorescence staining of cell cultures, HOK-16B cells were fixed with 4% paraformaldehyde and permeabilized with 0.2% Triton X-100 for 15 min each at room temperature, and blocked for 30 min with 2% BSA. Cells were subjected to immunofluorescence staining with anti-γH2AX (1:200, Thermo Fisher Scientific, cat no. A300-081A), HMGB1 (1:200, Abcam, cat no. ab18256) primary antibodies overnight at 4°C. Next day, cells were stained with Flamma®552-conjugated goat anti-rabbit IgG (BioActs) for 30 min. The fluorescence signals were visualized with an EVOS FL Auto Imaging System (Thermo Fisher Scientific).

For immunofluorescence staining of tissue sections, OLP tissues were fixed with 10% formalin at room temperature for 24 h and embedded in paraffin. Tissue slices, with a thickness of 4 µm, were then deparaffinized and rehydrated. After blocking samples for 2 h with a 10% BSA solution containing 4% normal goat serum (Vector Laboratories, Newark, CA, USA, cat no. S-1000), phospho-STAT3 (1:200, Cell signaling) and HMGB1 (1:200, Abcam) primary antibodies were applied. The slides were then incubated in the dark condition for 90 min with Alexa Fluor 488 or 555 (1:200, Abcam) and then stained with DAPI for 10 min. The fluorescence signals were visualized with an EVOS FL Auto Imaging System (Thermo Fisher Scientific).

### 2.5. Reverse transcription-quantitative (RT-q) PCR

Total RNA was extracted from HOK-16B cells by using a FavorPrep™ Blood/Cultured Cell Total RNA Kit (Favorgen Biotech Corporation, Taiwan), according to the manufacturer's instructions. 200 ng of total RNA was reverse transcribed using First Strand cDNA Synthesis Kit (Thermo Fisher Scientific) according to the manufacturer's instructions. RT-qPCR was performed on cDNA samples with the Luna® Universal qPCR Master Mix (New England BioLabs) using the Mic qPCR Cycler (bio molecular systems, Australia). mRNA levels were normalized to *hRPL32* mRNA level as a control and calculated according to the ΔΔCt method. The sequences of the forward and reverse primers are as follows: *hRPL32*: forward, 5′-GAAGTTCCTGGTCCACAACG-3′ and reverse, 5′-GCGATCTCGGCACAGTAAG-3′; *hTNFα*: forward, 5′-AGCCCATGTTGTAGCAAACC-3′ and reverse, 5′-TGAGGTACAGGCCCTCTGAT-3′; *hIL6*: forward, 5′-AGCCACTCACCTCTTCAGAACGAA-3′ and reverse, 5′-AGTGCCTCTTTGCTGCTTTCACAC-3′; *hIL8*: forward, 5′-GAGAGTGATTGAGAGTGGACCAC-3′ and reverse, 5′- CACAACCCTCTGCACCCAGTTT-3′*.*

### 2.6. Reactive oxygen species (ROS) detection

Intracellular ROS levels were monitored using the fluorogenic CellROX® Orange reagent (Thermo Fisher Scientific, cat no. C10443). CellROX reagent was added to cultured HOK-16B cells at a final concentration of 5 µM for 30 min. Nuclear DAPI staining was conducted with NucBlue Live ReadyProbes Reagent (Thermo Fisher Scientific, cat no. R37605) for 5 min. Fluorescent microscopic images were acquired using the EVOS FL Auto Imaging System (Thermo Fisher Scientific).

### 2.7. Analysis of cell viability

Cell viability was examined using D-Plus™ CCK cell viability assay kit (Dongin LS, cat no. CCK-3000) by measuring the absorbance at 450 nm, according to manufacturer's protocol. Absorbance values were normalized to those of the vehicle control, and the data were expressed as percentage viability.

### 2.8. Extracellular ATP measurement

To measure levels of extracellular ATP released from HOK-16B cells, supernatants were collected 48 h after treatment of PBS or epinephrine. Cleared supernatants of each condition were acquired by centrifugation, and then amount of ATP was measured with ATP Determination Kit (Thermo Fisher Scientific, cat no. A22066).

### 2.9. Statistical analysis

All statistical analyses were performed using GraphPad Prism software (version 9, San Diego, CA, USA). The unpaired two-tailed Student's t-test was used for experiments comparing two sets of data unless noted. All results are expressed as mean ± s.e.m. Differences were considered significant when *P < 0.05, **P < 0.01, and ***P < 0.001.

## 3. Results

### 3.1. High dose epinephrine shows cellular toxicity on keratinocytes.

**Figure 1.**
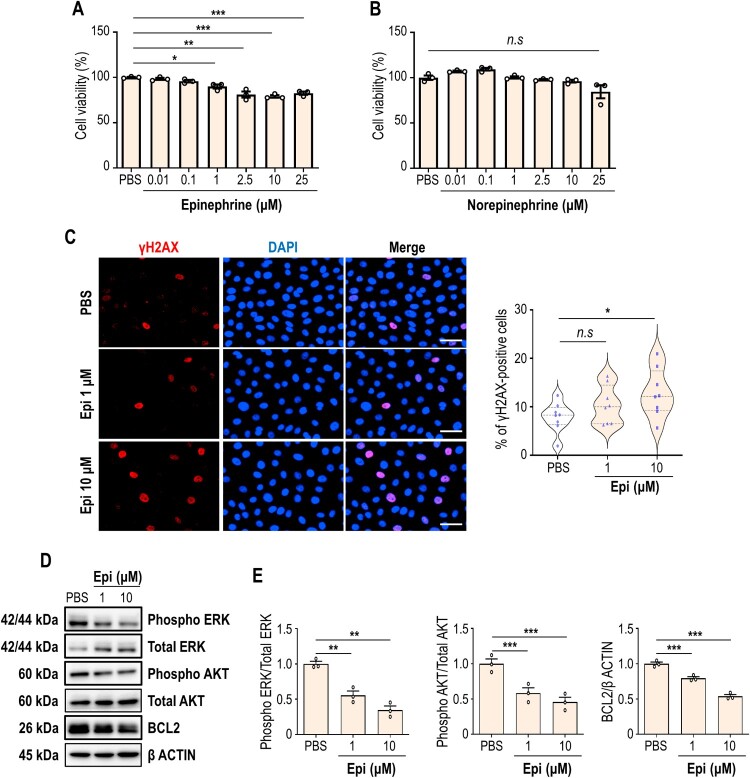
High level of epinephrine exhibits cytotoxicity on oral keratinocytes. (A and B) HOK-16B cells are maintained in the presence of 0.01, 0.1, 1, 2.5, 10, and 25 μM of epinephrine (A) or norepinephrine (B) for 72 h, and the cytotoxicity was determined by CCK cell viability assay. (C) HOK-16B cells were treated with vehicle or epinephrine (1 or 10 μM) for 24 h, and they were immunostained with a γH2AX antibody (red). Nuclear DAPI staining is presented in blue (left). The relative signal intensities of γH2AX are shown (right). Each symbol means an individual cell. (D) Immunoblot analysis of phospho and total ERK, phospho and total AKT, and BCL2 in HOK-16B cells treated with epinephrine for 24 h. β ACTIN was used as loading control. (E) Relative band intensities are shown. *P < 0.05; **P < 0.01; ***P < 0.001. *n.s*, not significant.

To find triggering factor in OLP pathogenesis, we tested the effect of catecholamines on oral keratinocytes. In physiological resting status, plasma catecholamine concentrations are low as 10^−10^∼10^−9^ M (Sluga et al. 2022). Surprisingly, however, acute maximal stress can result in a greater than 300-fold increase in the plasma catecholamine level. Indeed, some patients showed dramatic elevation of plasma epinephrine levels (up to 273 ng/ml ≈ 1.5 µM) under extreme stress condition (Wortsman et al. 1984). To evaluate the dose-dependent effect of catecholamine on cell physiology, HOK-16B oral keratinocytes were grown in the presence of epinephrine or norepinephrine, and the cytotoxicity was determined. When the concentration of epinephrine reaches 1 µM, the cell viability is significantly reduced to 90% and further decreases as the epinephrine concentration is increased (Figure 1A). In contrast, there is almost no change in cell viability when cells were treated with the concentration of norepinephrine up to 25 µM, indicating that norepinephrine has negligible effect on cell viability in this concentration range (Figure 1B).

Stress hormones were previously shown to induce DNA damage (Valente et al. 2021). To check the influence of epinephrine on DNA integrity in oral keratinocytes, the phosphorylation of γH2AX, a DNA damage marker, was tracked by immunofluorescence labeling. As a result of quantification, while the proportion of γH2AX-positive cells was not clearly increased by 1 µM epinephrine treatment, it was significantly increased when the concentration of epinephrine reaches 10 µM (Figure 1C), suggesting that high level of epinephrine induced DNA damage that leads to cell death at least in part. Inhibition of cell survival was additionally confirmed by immunoblotting. Phosphorylation of ERK and AKT, which generally promotes cell survival, was downregulated by epinephrine treatment (Figure 1D and 1E). Consistently, abundance of a pro-survival protein BCL2 was also reduced in the presence of epinephrine. Together, our data demonstrated that high concentrations of epinephrine show cytotoxicity on oral keratinocytes.

### 3.2. High dose epinephrine induces oxidative stress on oral keratinocytes.

Epinephrine can increase or decrease oxidative stress, depending on the situation (Alvarez-Diduk and Galano 2015; Djelic et al. 2019). If epinephrine causes cellular oxidative stress in oral keratinocytes, it can contribute to cytotoxicity shown above. In addition, recent GO enrichment analysis revealed that differentially expressed genes after chronic epinephrine exposure were mainly enriched in regards to oxidative stress-related pathway and the regulation of the cell death (Zhang et al. 2024).

**Figure 2.**
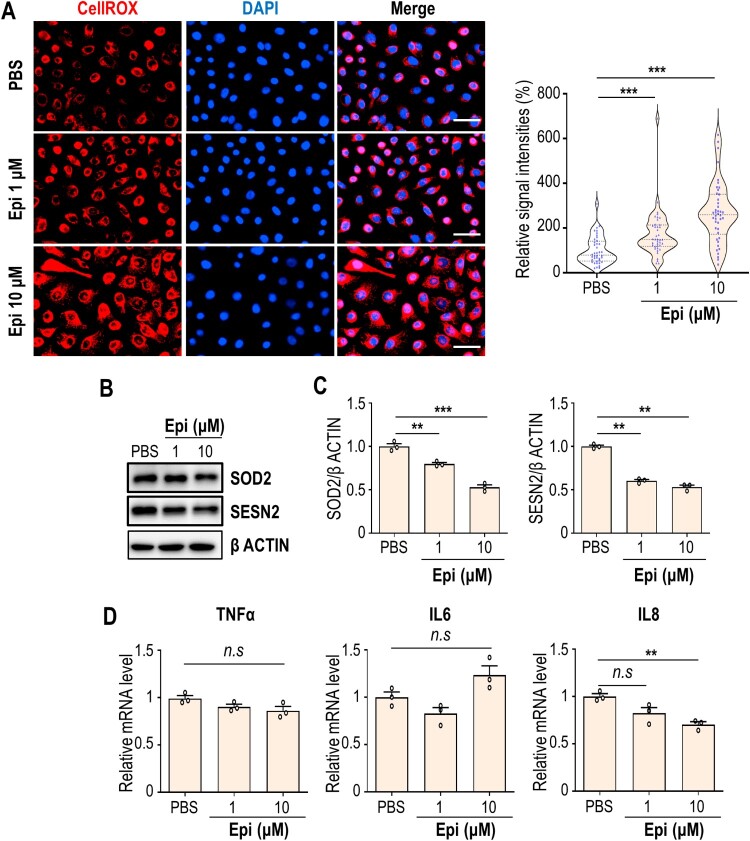
High level of epinephrine induces intracellular oxidative stress in oral keratinocytes. (A) HOK-16B cells treated with PBS or epinephrine for 24 h were subjected to CellROX staining. Nuclear DAPI signal is shown in blue (left). The relative CellROX signal intensities are shown (right). Each symbol means an individual cell. (B) Immunoblot analysis of SOD2 and SESN2 in HOK-16B cells treated with epinephrine for 24 h. β ACTIN was used as loading control. (C) Relative band intensities are shown. (D) Relative mRNA expression of TNFα, IL6, and IL8 in HOK-16B cells treated with epinephrine for 3 h. **P < 0.01; ***P < 0.001. *n.s*, not significant.

To assess the effect of epinephrine on oxidative stress in oral keratinocytes, ROS levels were determined by CellROX staining in vehicle or epinephrine-treated HOK-16B cells. As a result, ROS levels significantly increased following 1 or 10 µM epinephrine treatment (Figure 2A). Consistently, antioxidant protein SOD2 and SESN2 were markedly downregulated under the presence of epinephrine (Figure 2B and 2C), suggesting that high concentrations (≥ 1 µM) of epinephrine can reduce ROS scavenging potential of oral keratinocytes. Considering that oxidative stress and inflammation are linked as one could enhance the other, we examined levels of inflammatory cytokines that were induced in OLP lesion. Epinephrine failed to upregulate the mRNA expressions of TNFα and IL6, even at 10 µM. Rather, IL8 mRNA level was downregulated by 10 µM epinephrine treatment (Figure 2D). Together, epinephrine has a capacity to increase oxidative stress that may lead to the cytotoxicity on oral keratinocytes, without inflammatory activation.

Given that both alpha- and beta-adrenergic receptors are expressed in human keratinocytes (Yang et al. 2021), we further investigated their respective contributions to epinephrine-induced ROS generation. Treatment of HOK-16B cells with beta-adrenergic blockers (nadolol or timolol) or an alpha-adrenoceptor antagonist (yohimbine) markedly attenuated oxidative stress (Supplementary Fig. 1), suggesting that both receptor types contribute to epinephrine-induced ROS production.

### 3.3. High dose epinephrine activates STAT3 pathway in oral keratinocytes.

**Figure 3.**
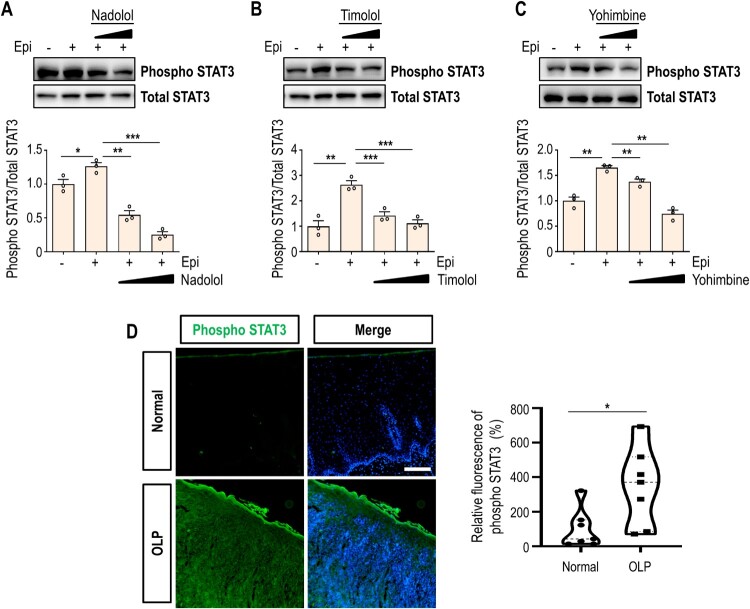
High level of epinephrine induces STAT3 phosphorylation in oral keratinocytes. (A-C) Immunoblot analysis of phospho STAT3 in HOK-16B cells treated with 10 μM epinephrine for 24 h, with or without Nadolol (A), Timolol (B), or Yohimbine (C). Two concentrations (1 or 10 μM) of each drug were used. Total STAT3 was used as loading control. Relative band intensities are shown in lower panels. (D) Normal and ulcerative-type OLP samples were immunostained with phospho STAT3 antibody (green). Nuclear DAPI staining is shown in blue. Scale bar, 200 μm. *P < 0.05; **P < 0.01; ***P < 0.001.

Oxidative stress is known to activate STAT3 through phosphorylation and other post-translational modifications. Intracellular ROS promote phosphorylation of STAT3 on tyrosine residues, facilitating its nuclear translocation (Carballo et al. 1999). To determine whether high-dose epinephrine induces STAT3 phosphorylation, we performed an immunoblot assay. As shown in Figure 3A–3C, exposure to high concentrations of epinephrine upregulated STAT3 phosphorylation. Clearly, treatment of HOK-16B cells with alpha- or beta-adrenergic blockers effectively reversed STAT3 hyperphosphorylation, suggesting that both receptor types contribute to epinephrine-induced STAT3 activation (Figure 3A-3C). Consistent with these findings, phosphorylated STAT3 levels were significantly elevated in OLP lesions compared to non-OLP controls (Figure 3D).

To determine whether high-dose epinephrine-induced cytotoxicity is mediated through adrenergic receptor signaling, cells were treated with cirazoline (alpha 1A agonist), clonidine (alpha 2 agonist), or isoproterenol (non-selective beta agonist). All agonists elicited robust STAT3 phosphorylation at high concentrations (≥ 10 μM) (Supplementary Fig. 2A), mirroring the response to high-dose epinephrine, and hyperactivation of either alpha- or beta-adrenergic receptors consistently triggered oxidative stress (Supplementary Fig. 2B).

### 3.4. Epinephrine increases HMGB1 level and ATP release in oral keratinocytes.

**Figure 4.**
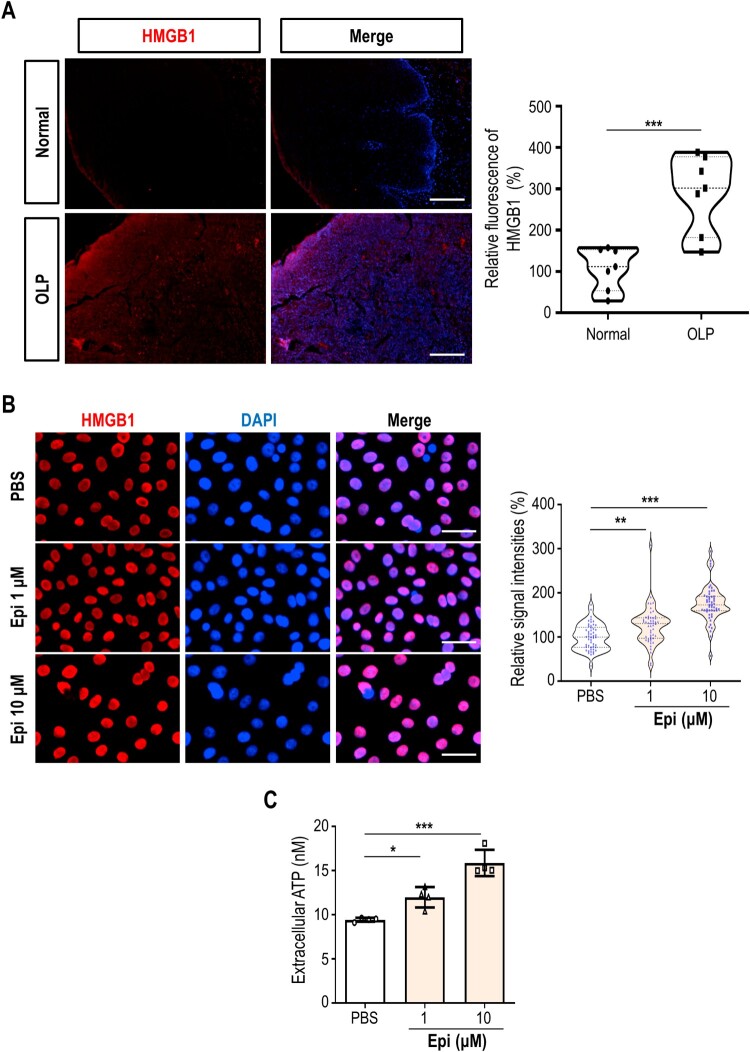
High level of epinephrine induces cell death in oral keratinocytes. (A) Normal and ulcerative-type OLP samples were immunostained with HMGB1 antibody (red). Nuclear DAPI staining is shown in blue. Scale bar, 200 μm. (B) HOK-16B cells were treated with vehicle or epinephrine (1 or 10 μM) for 24 h, and they were immunostained with a HMGB1 antibody (red). Nuclear DAPI staining is presented in blue (left). The relative signal intensities of HMGB1 are shown (right). Each symbol means an individual cell. (C) HOK-16B cells were treated with vehicle or epinephrine (1 or 10 μM) for 48 h, and levels of extracellular ATP were measured by bioluminescence assay. *P < 0.05; **P < 0.01; ***P < 0.001.

During cell death, molecular signals called damage-associated molecular patterns (DAMPs) can be induced and released from cells. DAMPs are implicated in immunogenic cell death by activating immune cells, and modulate the chronicity of the inflammatory process. We first checked the level of HMGB1, one of the major DAMPs that triggers inflammatory disease progression, in OLP lesions. Clearly, immunofluorescence staining showed that HMGB1 level was significantly enhanced in OLP epithelium, compared with non-OLP tissues (Figure 4A). To examine the influence of epinephrine on DAMPs production, HMGB1 was tracked by immunofluorescence labeling. As a result of quantification, HMGB1 abundance was significantly augmented by 1 or 10 µM epinephrine treatment (Figure 4B). We further measured the level of secreted ATP that has been regarded as the ‘find me’ signal to recruit immune cells (Solari et al. 2020). ATP secretion to the extracellular medium was moderately increased by 1 or 10 µM epinephrine treatment (Figure 4C). Collectively, our results suggest that the cytotoxicity mediated by high concentrations (≥ 1 µM) of epinephrine can lead to the DAMP production and release.

## 4. Discussion

In this study, we suggest that epinephrine may have a promoting role in OLP pathogenesis. Although the concentration of epinephrine has not been precisely monitored in OLP lesions so far, several previous works support our hypothesis: (1) a major metabolite of epinephrine, 3,4-dihydroxymandelic acid, is highly upregulated in patients with OLP (Yang et al. 2020), (2) plasma epinephrine level can be clinically increased up to 1.5 µM (Wortsman et al. 1984) (epinephrine showed cytotoxicity at concentrations above 1 µM, according to our results), (3) catecholamine-mediated beta adrenergic receptor signaling activation induces ROS accumulation followed by cell death (Sun et al. 2015), and (4) catecholamines may be the first ‘danger signal’ to immune system and stimulate immune response (Ortega et al. 2007). Considering that a rich vascular proliferation has been detected in OLP (Scardina et al. 2009), the impact of epinephrine can be greatly amplified in the OLP lesions by enhanced angiogenesis.

The cytotoxicity induced by high-dose epinephrine in keratinocytes suggests that stress may serve as a critical trigger for epithelial cell damage in OLP. Given that OLP is a chronic disease with intermittent flare-ups, our findings align with the hypothesis that psychological stress and acute epinephrine surges may contribute to the timing of OLP exacerbations. The stress-mediated damage to keratinocytes might initiate a cascade of immune activation, leading to sustained inflammation and further tissue damage. In addition, oxidative stress, which we demonstrated to be significantly increased in keratinocytes treated with high doses of epinephrine, may be a key factor driving OLP pathology. Previous studies have implicated oxidative stress as a contributor to the immune dysregulation observed in OLP, linking ROS accumulation to T cell-mediated cytotoxicity (Shiva et al. 2020). Thus, our findings provide a mechanistic link between stress, oxidative stress, and epithelial damage in OLP progression.

While upregulation of HMGB1 was previously reported in skin lesions of lichen planus, research on DAMPs has so far been of little interest in the OLP. We found that HMGB1 level was significantly increased in the epithelium of ulcerative-type OLP lesions. In addition, we showed that epinephrine was able to increase HMGB1 level and ATP release in HOK-16B cells. However, the presence and concentration of released ATP and other DAMPs were not additionally investigated in the present study. Given the importance of DAMPs in immunogenic cell death, the relevance of DAMPs in OLP onset and progression needs to be further explored.

Research on DAMPs has so far been of little interest in the OLP. We found that HMGB1 level was significantly increased in the epithelium of ulcerative-type OLP lesions. In addition, we showed that epinephrine was able to increase HMGB1 level and ATP release in HOK-16B cells. However, the presence and concentration of released ATP and other DAMPs were not additionally investigated in the present study. Given the importance of DAMPs in immunogenic cell death, the relevance of DAMPs in OLP onset and progression needs to be further explored. The contribution of DAMPs in immune activation is particularly intriguing, as OLP is characterized by a persistent inflammatory infiltrate dominated by CD8+ T cells. It remains to be elucidated whether stress-induced DAMP production can directly modulate T cell activation in OLP lesions. Additionally, future studies should explore whether blocking the effects of DAMPs could be a therapeutic strategy for mitigating OLP progression.

In addition to apoptosis, dyskeratosis represents another hallmark of epithelial pathology in OLP. Histopathological and molecular evidence suggests that OLP epithelium undergoes aberrant keratinocyte differentiation characterized by premature keratinization (Bloor et al. 2000). Although our present study focused on the cytotoxic effects of epinephrine in oral keratinocytes, the potential influence of epinephrine on differentiation-related markers, such as keratin 10, involucrin, and filaggrin, warrants further investigation. Considering that dyskeratosis can compromise epithelial barrier integrity (Danielsson et al. 2014; Shimada et al. 2018), such analyses may elucidate an additional mechanism by which epinephrine contributes to OLP progression. Moreover, catecholamines including epinephrine have been reported to modulate keratinocyte differentiation through adrenergic receptor-mediated signaling (Schallreuter et al. 1995). Future studies employing both monoculture and co-culture systems with immune or stromal cells will be essential to comprehensively understand the interplay between cytotoxicity, differentiation abnormalities, and chronic inflammation in OLP.

Our study also monitored the STAT3 activation in OLP pathogenesis. Given that ROS have negligible effect on the translocation of STAT1 or STAT5B into the nucleus, the interplay between ROS and STAT3 seems rather specific and highlights the role of STAT3 in cellular responses to oxidative damage (Carballo et al. 1999). Furthermore, STAT3 signaling is involved in cell fate decision and immune regulation (Grusanovic et al. 2023), its upregulation in OLP epithelium suggests a potential role in maintaining chronic inflammation. STAT3 is known to facilitate immune cell recruitment (McLoughlin et al. 2005), which could further amplify tissue damage. However, the precise role of STAT3 in OLP remains to be fully elucidated. Future studies should investigate whether targeting STAT3 could serve as a therapeutic approach for OLP patients, particularly those with stress-related exacerbations.

Although our study provides novel insights into the relationship between epinephrine and OLP, there are some limitations. First, our experiments were conducted using an in vitro keratinocyte model, which does not fully replicate the complex tissue microenvironment of OLP lesions. The interplay between epithelial cells, immune cells, and stromal components in OLP needs to be further examined in in vivo models. Second, while we demonstrated that high-dose epinephrine induces oxidative stress and DAMP release, the downstream effects on immune activation remain unclear. Further research is required to determine how catecholamine-induced oxidative stress influences immune responses in OLP patients.

Our findings support a model in which epinephrine, particularly under high-stress conditions, binds to both alpha- and beta-adrenergic receptors expressed on keratinocytes. Receptor engagement leads to activation of intracellular signaling cascades that converge on the generation of ROS, as evidenced by the downregulation of antioxidant proteins SOD2 and SESN2. Elevated ROS appears to act as a key upstream signal for STAT3 activation, since oxidative stress is known to promote STAT3 tyrosine phosphorylation and nuclear translocation. Activated STAT3 can then modulate transcription of genes involved in stress responses, survival, and inflammation. In parallel, ROS and STAT3 activation together may contribute to the upregulation and release of DAMPs such as HMGB1 and ATP. These DAMPs can act as extracellular ‘danger signals,’ capable of recruiting and activating immune cells, thereby amplifying the inflammatory milieu in OLP lesions. This hierarchical framework integrates our observations into a coherent mechanistic pathway and underscores the potential for therapeutic intervention from receptor blockade to antioxidant therapy or STAT3 inhibition. These findings provide a mechanistic explanation for the well-documented association between psychological stress and OLP exacerbations. Future research should focus on exploring potential therapeutic strategies targeting stress-related pathways in OLP treatment.

## Supplementary Material

Supplementary Figures
