# Supplementary figures and images for: Epinephrine as a potential driver of oral lichen planus pathogenesis
Source: Anim Cells Syst (Seoul). 2025 Nov 17;29(1):653–64. doi: 10.1080/19768354.2025.2588914 (PMC12624973; doi:10.1080/19768354.2025.2588914)

Supplementary Figure 1

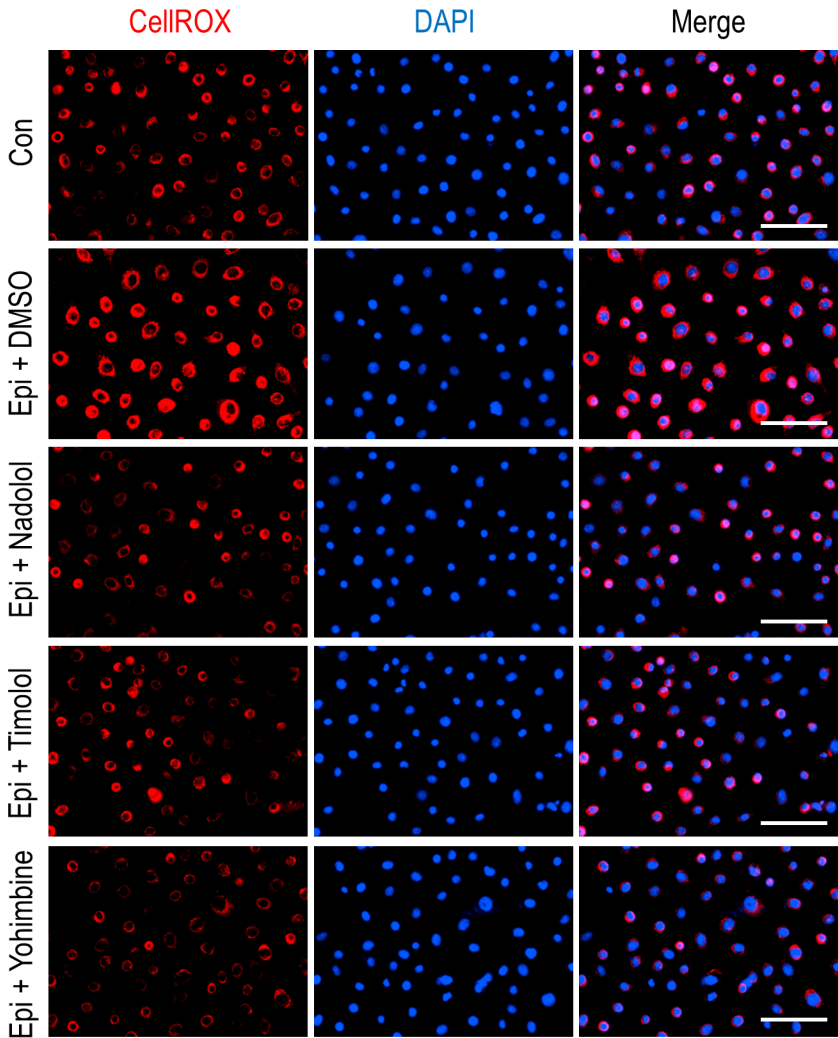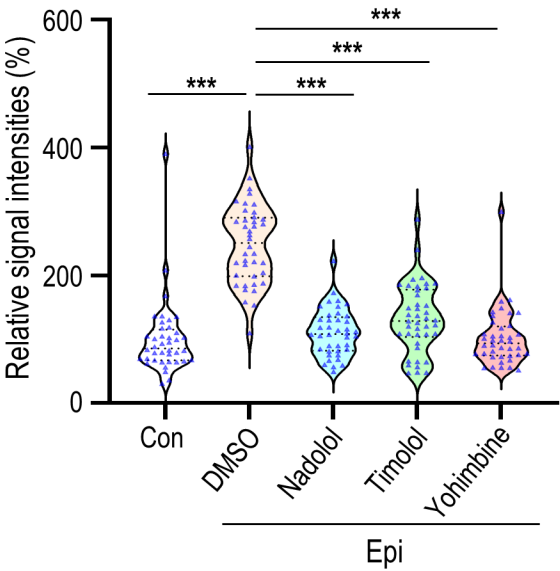

Supplementary Figure 2

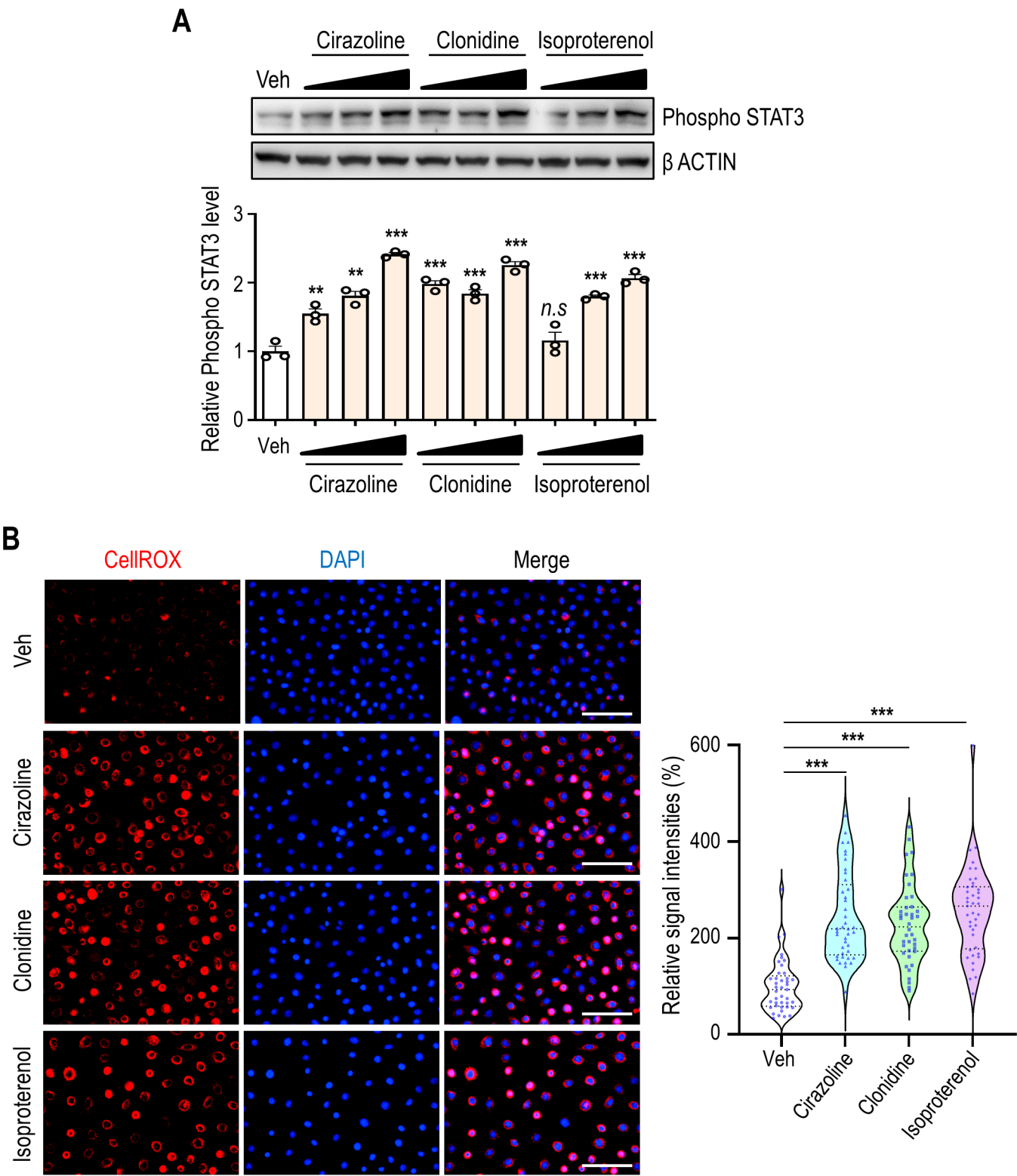

Supplement: Supplementary Figures [file TACS_A_2588914_SM6563.pdf]
